# Tightly binding valence electron in aluminum observed through X-ray charge density study

**DOI:** 10.1038/s41598-018-30470-1

**Published:** 2018-08-10

**Authors:** Tomoaki Sasaki, Hidetaka Kasai, Eiji Nishibori

**Affiliations:** 1 Graduate School of Pure and Applied Sciences, University of Tsukuba, Tsukuba, 305-8571 Japan; 2 Faculty of Pure and Applied Sciences and Tsukuba Research Center for Energy Materials Science (TREMS), University of Tsukuba, Tsukuba, 305-8571 Japan

## Abstract

Accurate and high reciprocal resolution experimental structure factors of aluminum were determined from a synchrotron powder X-ray diffraction data measured at 30 K with sin *θ*/*λ* < 2.31 Å^−1^. The structure factors have small deviations from independent atom model in sin *θ*/*λ* < 0.83 Å^*−*1^. Theoretical structure factors were prepared using density functional theoretical calculations by full potential linearized augmented plane wave method. The deviation between experimental and theoretical data was also observed at around sin *θ*/*λ* ≈ 0.4 Å^−1^. The charge density was determined by an extended Hansen-Coppens multipole modeling using experimental and theoretical structure factors. Charge density maxima at tetrahedral site were observed in both experimental and theoretical deformation density. The charge-density difference peaks indicating directional bonding formation were observed in the difference density between experiment and theory. The present study reveals tight binding like character of valence electron of aluminum. The fact will provide a crucial information for development of high-performance aluminum alloy.

## Introduction

Metal is one of the oldest materials used in human history. We would like to know an origin of function of metal for a long time. Historically important works were carried out by P. K. L. Drude and H. A. Lorentz in the 19th century^1^. An electron theory of metals has been established by N. F. Mott and H. Jones, etc. at around 1930 soon after an establishment of quantum mechanics. Pure metals and simple alloys were the central topics in the first stage of quantum condensed matter physics. An extraordinary amount of research including electronic properties and structure have been carried out during the past 100 years.

Nearly free electron model and tight binding model are two kinds of extremely simple approximation for solids in theoretical study. Nearly free electron model is appropriate for simple metal system such as aluminum and copper. Tight binding model is appropriate for covalent bonding materials such as silicon. The mechanical properties of metals were relatively recently explained based on quantum mechanics by the first principle calculation in the beginning of the century using the calculation power of computer^2–5^. The relationship between pressure-hardening in aluminum and directional bonding was elucidated by the theoretical calculation^2^. High quality charge-density distributions of metal by the theoretical calculation were also used for the estimation of mechanical properties. Eberhart and Jones reported that the coefficient of determination *R*^2^ = 0.975 in regression analysis was found between theoretical and experimental Cauchy pressure in pure tungsten, molybdenum, tantalum, vanadium, and niobium^5^, using a topological analysis of charge density^6^.

Charge density studies of metal have been carried out for more than 40 years using X-ray diffraction (XRD). These studies showed the difficulty of observation of chemical bonding in metal system. The contribution of bonding electron in diffraction data is lower than those of organics and semiconductors. Their diffraction patterns often have contribution of thermal diffuse scattering owing to the low Debye temperature of metals. In addition, the structures of metal often include intrinsic strain and stacking fault. These facts prohibit to measure the high-quality diffraction data for charge density study.

Nakashima *et al*. practically solved the difficulty to measure extremely accurate low order structure factor using quantitative convergent-beam electron diffraction (QCBED) technique^7^. They measured 111 and 200 reflections of aluminum with sufficient accuracy to detect the deviations from free electron gas model. The charge density distribution of aluminum has been determined using the combined data of QCBED and XRD. They observed electron accumulation at tetrahedral site which is consistent with the density functional theory. They also found the relationship between Young’s modulus and observed deformation density.

Quantitatively accurate charge density of metal provides an important clue for understanding, development, and improvement of metal and related alloys^7^. Recent progress of synchrotron radiation (SR) XRD for charge density study enables us to perform such type of study. The diffractometers and measurement techniques of SRXRD were developed during the past decade^8–10^. Very small amounts of electron distributions were successfully observed in TiS_2_^11^ and LaB_6_^12^ using the diffractometers and techniques. The method can be applied to pure metal system. In this study, we did accurate charge density study of aluminum using the state of the art SRXRD.

We selected aluminum as the sample of the present study, since aluminum is one of the ideal metals to determine the accurate experimental charge density. Ogata *et al*. reported the high intrinsic stacking fault energy by theoretical calculation^2^. We can measure diffraction data without contribution from stacking fault. In addition, aluminum has relatively large contribution of valence electron to diffraction data in metal. Bonding electron density of aluminum based on the Drude model is the second largest value, 0.18 eÅ^−3^, in the typical metals^1^. Aluminum is widely used in industry as aluminum alloy.

## Results

Multi-temperature overlaid powder diffraction profiles^8^ of aluminum were measured at SPring-8 BL02B2 beamline. The sample temperatures were controlled at 30, 100, 200, 300, 400, 500, and 600 K using gas flow devices. High-energy X-ray with 37 keV was used to reduce effects of absorption and extinction. The multi-temperature dataset was used to estimate thermal effects including anharmonic thermal vibrations and thermal diffuse scattering in diffraction data. We found thermal effects could be negligible in low temperature data below 100 K.

The powder profiles were analyzed by the combination of Rietveld refinement and multipole refinement. The details of analysis were described in elsewhere^12^. The observed structure factors were extracted from 30 K data. Total number of the structure factors is 217 which corresponds to sin *θ*/*λ* < 2.31 Å^−1^ reciprocal resolution. We also prepared three sets of theoretical structure factors. Two of three were prepared by WIEN2k program^13^ using two kinds of exchange parameters Perdew–Burke–Ernzerhof generalized gradient approximation (PBE) and local spin density approximation (LSDA). The structure factors from independent atom model (IAM) were also prepared using XD2016 program^14^. The reciprocal resolution of these data is the same as that of the experimental data.

**Figure 1:**
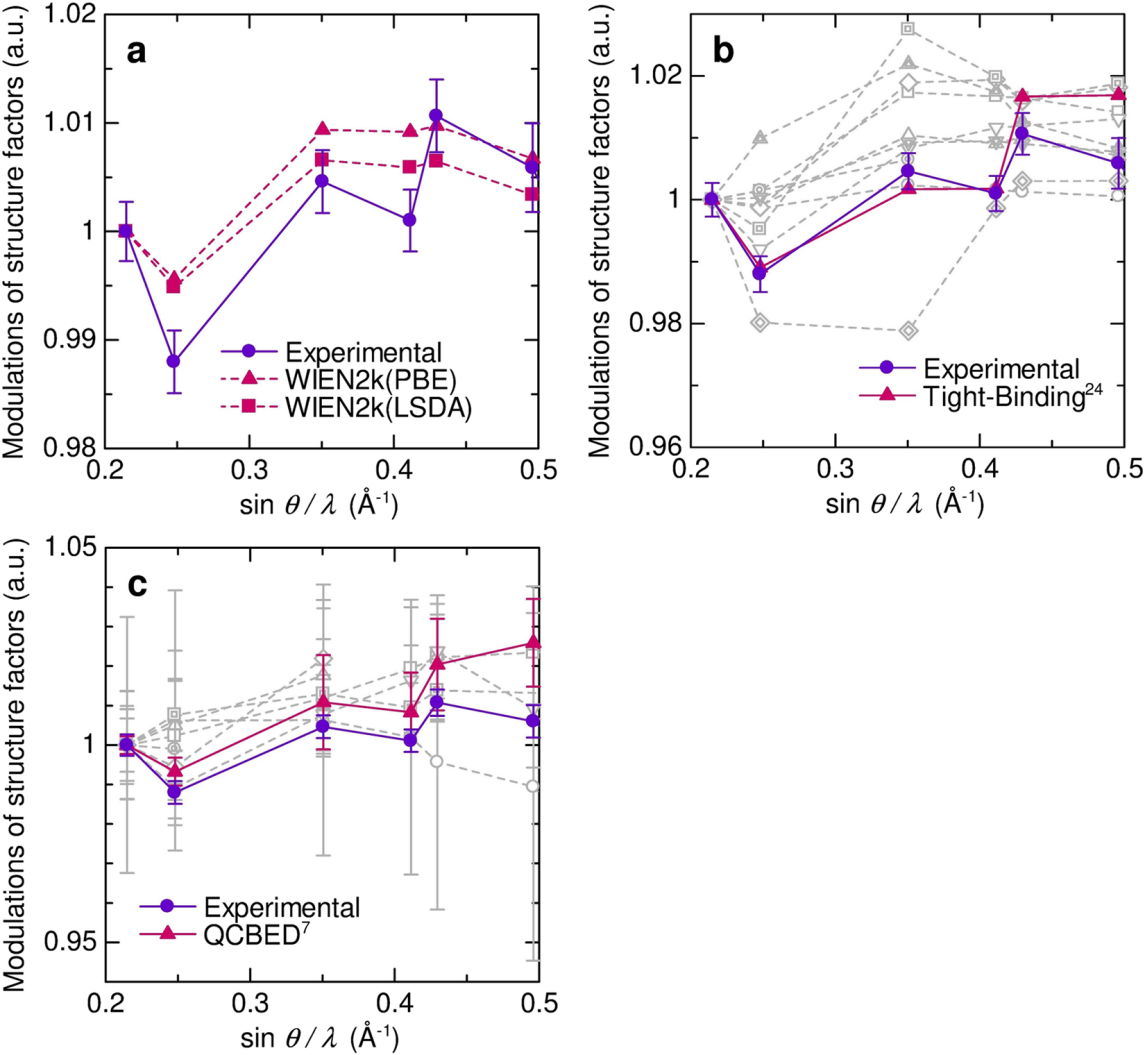
Relative ratio of structure factors to IAM normalized by 111 reflection. Horizontal axis is reciprocal resolution sin *θ/λ*. (**a**) The present experimental and theoretical values. (**b**) Reported theoretical values and the present experimental values. Gray lines, which represent the reported theoretical values in refs^15–24^, show no agreement with the present experimental values. (**c**) Reported experimental values and the present experimental values. Gray lines have no significant deviation from 111.

Figure 1a shows plots of relative ratio of structure factors to IAM for the present experimental data and theoretical values. The deviation from IAM in the lowest three reflections of the experimental data are almost identical to those of theoretical values. The plot of deviations after the fourth reflections in theoretical data are almost flat, whereas the experimental data after the fourth reflection still have some fluctuation. Figure 1b shows the ratio of reported theoretical data in the literatures^15–23^. The ratio of tight-binding approximation^24^ shows similar fluctuations to the experimental data including after fourth reflections.

Many sets of observed structure factors for aluminum have been reported so far. Figure 1c is the plot of their observations^7,25–31^. The first three reflections of QCBED data show similar deviation to the present experiment and theoretical calculations. The others cannot recognize the deviation from unity. Those can be regarded as unity within experimental errors. Aluminum is one of the ideal metals to determine the accurate experimental charge density owing to relatively high valence electron ratio. The present study shows the precisions of the QCBED and the state of the art SRXRD are required to detect charge density modulation from IAM.

The charge density distribution of aluminum was determined by the multipole refinement of the experimental and theoretical structure factors. A multipole model including core electron deformation terms was used in the analysis. The reliability factors of multiple refinement were 1.97%, 0.08%, and 0.10% for the experimental data, WIEN2k PBE, and WIEN2k LSDA, respectively. The determined static deformation density of WIEN2k PBE is almost identical to WIEN2k LSDA as shown in Supplementary Note and Supplementary Fig. S1. We used the result of WIEN2k PBE in the following discussions.

**Figure 2:**
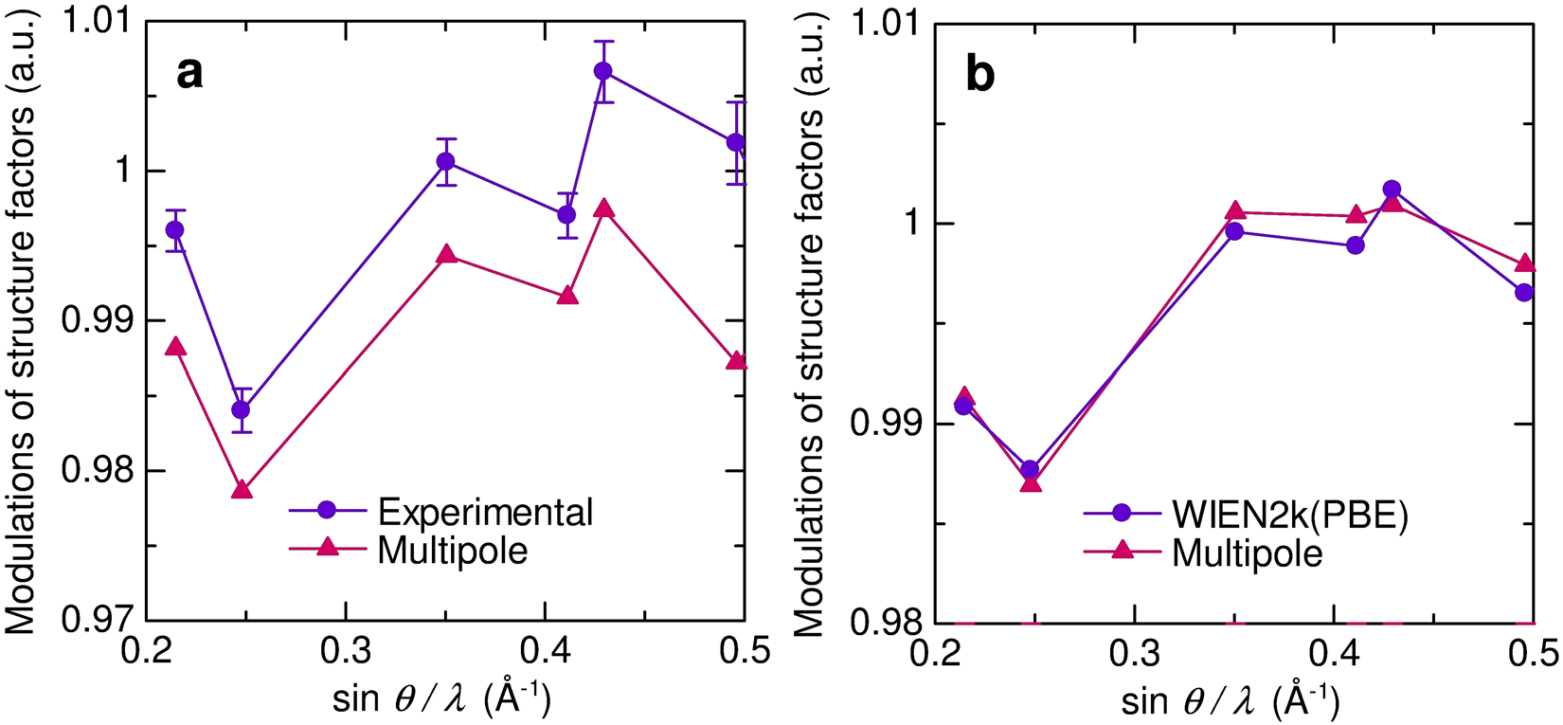
Relative ratio of structure factors to IAM. Horizontal axis is reciprocal resolution sin *θ*/*λ*. (**a**) The present experimental and multipole model values. (**b**) The present theoretical and multipole model values.

Figure 2a shows plots of relative ratio of structure factors to IAM for the multipole model and experimental data. The deviations of both the first three and after fourth were clearly expressed by the multipole model. Figure 2b shows plots of relative ratio of structure factors to IAM for the multipole model and theoretical structure factors. The deviations of the first three were well represented by the multipole modeling. Small deviations were found after fourth of the multipole model. The multipole modeling is an expansion using atom-centered spherical harmonics. The model can well express the charge density by the combination of atomic wave function such as tight-binding method. The deviations after fourth reflection in the experimental data indicate that the charge density of aluminum has tight-binding like character.

**Figure 3:**
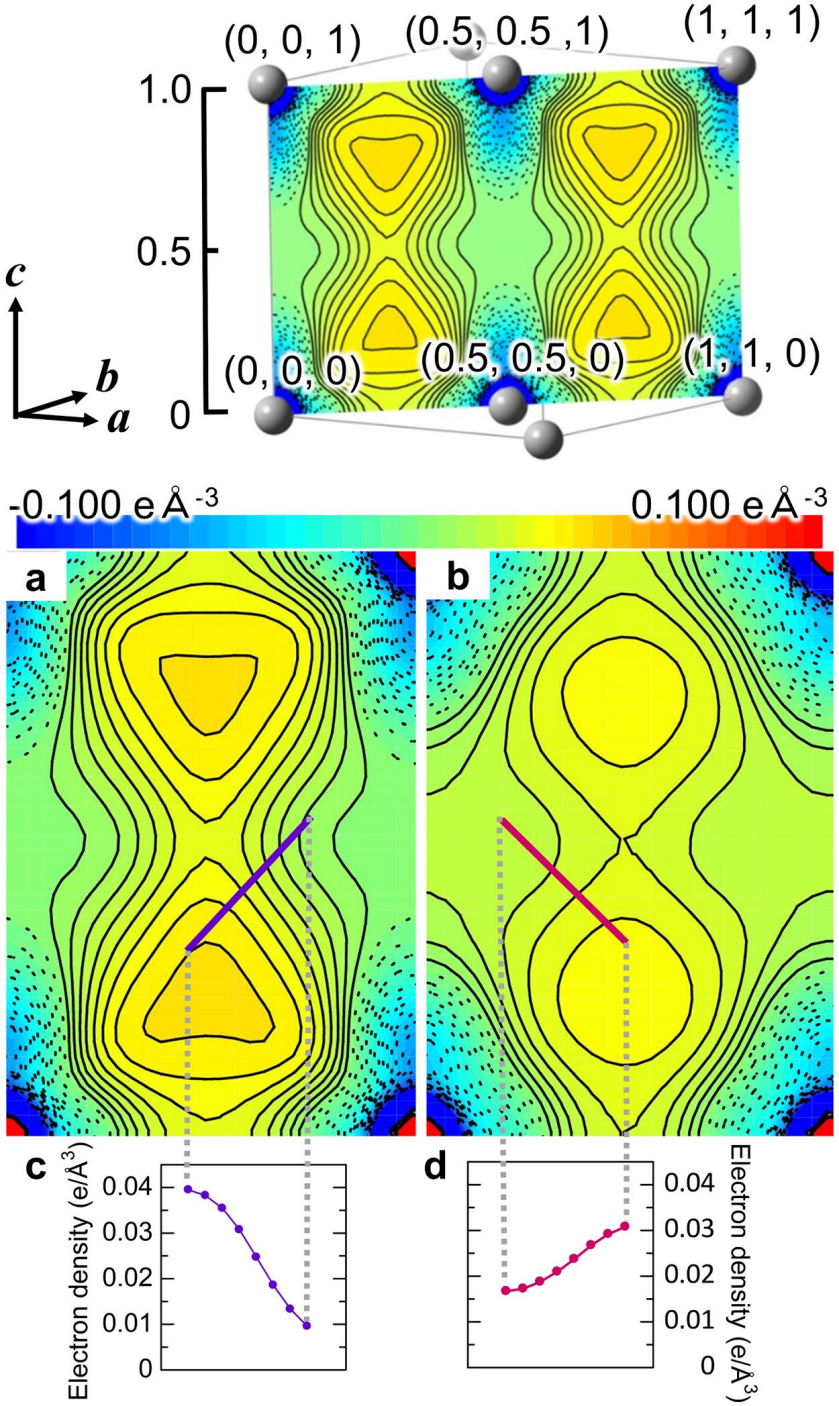
Static deformation density of (110) plane. The present experimental (**a**) and theoretical deformation density (**b**). The drawing (110) plane is shown in the upper inset. Aluminum atoms are at four corners. The contour interval is 0.005 eÅ^−3^ in −0.1–0.1 eÅ^−3^ region. Solid and dotted lines show positive and negative contours, respectively. (**c**,**d**) Electron density values along purple and red solid line in (**a**) and (**b**), respectively.

Figure 3a,b show static deformation density of (110) plane for experiment and theory. The deformation density shows maxima at the center of tetrahedral site which is consistent with the previous study^7^. Figure 3c,d also show the one-dimensional charge density along lines on the deformation density map. The charge density at the peak maxima are 0.04 and 0.03 eÅ^−3^ for experiment and WIEN2k PBE, respectively. A number of electrons in the peaks are ~0.06 e and ~0.03 e for experiment and WIEN2k, respectively. Very small amount of accumulation was successfully observed from SRXRD. Wide spread electrons in left-right direction were found in Fig. 3b. The charge density at octahedral site of experiment is 0.01 eÅ^−3^ smaller than those of theories.

## Discussion

Nakashima *et al*. reported that tetrahedrally centered interatomic bonding could connect Young’s coefficients of aluminum^7^. The bonding observed in the present study is also the strongest along [111] and weakest along [100] among [100], [110], and [111] directions. Young’s modulus, *E*_uvw_, is proportional to Δ*ρ*, *E*_100_ < *E*_110_ < *E*_111_. These facts are also consistent with the present study. Charge density of experiment is more localized at the center of tetrahedral than theoretical result. This fact is also consistent with the result of QCBED.

**Figure 4:**
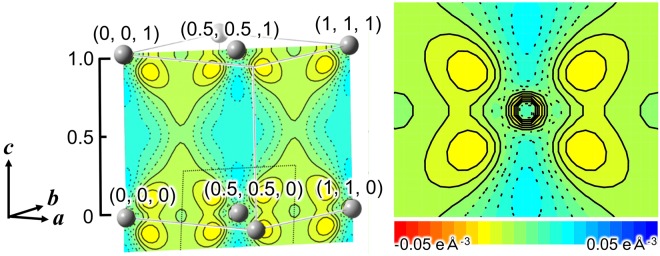
Charge-density difference between experiment and theory. The drawing plane is indicating in the left inset inside black dotted line. Aluminum atom is at the center of the plane. The contour interval is 0.005 eÅ^−3^ in −0.05–0.05 eÅ^−3^ region. Solid and dotted lines show positive and negative contours, respectively.

The previous QCBED study had never described an origin of the higher charge density than theory at tetrahedral site. The present result also shows the higher charge density than theoretical one. To reveal the origin of the density we made charge-density difference between experiment and theory. Figure 4 shows the map of charge-density difference. We can recognize peaks around atomic sites that is similar to atomic orbital. This fact suggests that the deviation of structure factors after fourth reflections in Fig. 1b indicates the existence of isolated atomic like orbital. Superposition of wave functions for isolated atoms located at each atomic site was used in the tight-binding model. The present result indicates that charge density of aluminum has small amounts of tight binding like character supported from Figs 1a, 2a and 4. The fact provides a new view of the metallic bonding of aluminum since the nearly free electron model is considered as the good approximation. In addition, the structure factors after fourth reflection were required to reveal the tight-binding like feature which have never been observed with sufficient accuracy so far. QCBED method can measure extremely accurate low order structure factors. The weak point of the method is there is blind region. The advantage of SRXRD is to measure full resolution data set.

Aluminum alloy in which aluminum is the predominant metal is widely used in industrial applications of many fields. Quantitatively estimation of the change of chemical bonding for doping will provide the most important knowledge to design, develop, and synthesis of high-performance aluminum alloy. It is difficult to estimate the change from theoretical calculation since treatment of dopant is not easy task for theoretical study. X-ray charge density study can be applied to the alloy system. High resolution charge density study of aluminum alloy will improve manufacture process of aluminum alloy.

Nowadays, third generation SR facilities can be used in the almost every advanced country. Similar quality of SRXRD can be measured elsewhere in the world. Charge density distribution in the material is crucial to understand their properties based on quantum mechanics. X-ray charge density is the most information rich observables in natural science. Technique used in the present study can be applied to many kinds of metals. We will perform charge density studies of metals using present technique. The correlations between mechanical properties and charge density of metals will be revealed in the near feature.

## Methods

### Sample preparation

Aluminum powder with 99.9% purity and 3 μm average particle size was used as a sample. The powder was sealed in a glass capillary with argon gas. The internal diameter of the capillary is 0.4 mm. A glovebox was used for sample preparation under argon atmosphere. The capillary was sealed by a fire.

### Synchrotron powder X-ray diffraction

Synchrotron Radiation X-ray powder diffraction experiments were carried out at SPring-8 BL02B2 beamline. A large Debye-Sherrer camera with an Imaging Plate (IP) as a detector was used for data collection. The size of IP was 200 × 400 mm. The sample was rotated during measurement to reduce effect of preferred orientation. Powder data at 30 K was measured using a He gas flow low temperature device. Powder profiles at 100, 200, and 300 K were measured using a N_2_ gas flow low temperature device. The wavelengths of incident X-ray were 0.32895(1) Å for 30 K data, and were 0.32862(1) Å for 100, 200, and 300 K. The wavelengths of incident X-ray were calibrated by the lattice constant of NIST CeO_2_ standard sample. The angular dependence of absorption between 10° and 100° in 2*θ* is 0.102%. Two powder profiles were measured at each temperature point to improve the reciprocal resolution and counting statistics of high order data^32^. The 2*θ* range of first and second data were from 0 to 78° and from 30 to 108° with 0.01° step which corresponds to *d* > 0.261 Å and 0.635 > *d* > 0.203 Å in *d*-spacing range. The exposure time of the first and second data were 27 min and 108 min at 30 K. The exposure time of the first and second were 30 min and 120 min at 100, 200, and 300 K. Two kinds of one-dimensional powder profiles were created from one two-dimensional image by the integration with 51 pixels and 251 pixels in the perpendicular direction of 2*θ*.

### Data analysis by Rietveld refinement

Rietveld refinements using the multiple datasets were carried out using program Synchrotron Powder (SP)^8^. The reciprocal resolution in the analysis are corresponding to sin *θ*/*λ* < 2.31, 2.15, 1.77, and 1.62 Å^−1^ for 30, 100, 200, and 300 K data. The observed structure factors were initially extracted from the results of Rietveld refinements based on IAM. The extraction of the observed structure factors was improved by an iterative procedure of multipole refinement and powder diffraction pattern fitting. The reliability factors based on weighted profile *R*_wp_ of the final pattern fitting were 1.87%, 1.89%, 2.41%, and 2.73% for 30, 100, 200, and 300 K, respectively. The final reliability factors based on Bragg intensity *R*_I_ were 2.37%, 2.85%, 3.19%, and 3.13% for 30, 100, 200, and 300 K. The determined lattice constants, *a*, isotropic atomic displacement parameter, *U*_iso_, and their temperature dependence are shown in Supplementary Table S1 and Fig. S2.

### Theoretical calculation of charge density

Supplementary Table S2 shows the theoretical structure factors *F* of aluminum in sin *θ*/*λ* < 2.31 Å^−1^. First principle calculation based on density functional theory was performed by full potential-linearized augmented plane wave (FP-LAPW) with the generalized gradient approximation (GGA), local orbitals, and local screening potentials in WIEN2k package^13^. Experimental lattice constants were used for the calculations. We used 1000 k points with plane-wave cutoff parameter *R*_MT_*K*_max_ = 7.0. Theoretical structure factors were calculated by lapw3 program.

### Multipole modeling of theoretical structure factors

Supplementary Table S3 shows multipole parameters by XD2016^14^ for the theoretical structure factors. Extended Hansen-Coppens multipole model including core deformation term^33^ was used for the analysis. The electron configuration of aluminum was 1 *s*^2^ 2 *s*^2^ 2*p*^6^ 3 *s*^2^ 3*p*^1^ ^34^. We set 1 *s*^2^, 2 *s*^2^ + 2*p*^6^, and 3 *s*^2^ + 3*p*^1^ valence electron shells. The local axis for aluminum atom were parallel to [100], [010], and [001] directions. The structure factor from IAM were also prepared using XD2016 program. Scale factor *s*, radial expansion/contraction parameters, *κ*_core_ and *κ*_valence_, and hexadecapole parameters, H0, were refined in the analysis. There is relationship between H0 and H4 + , H4 +  = 0.74048H0.

### The estimation of first order thermal diffuse scattering in diffraction data

Supplementary Fig. S3a shows first order thermal diffuse scattering of aluminum by Herbstein’s equation^35^ for 30, 100, 200, and 300 K. Horizontal axis is diffraction angle 2*θ*. We estimated amounts of thermal diffuse scattering using calculated profiles. Supplementary Fig. S3b shows first order thermal diffuse scattering at 30 and 300 K together with intensity baselines colored by purple and green. Supplementary Fig. S3c shows the modulations of calculated first order thermal diffuse scattering from the baselines. Horizontal axis is diffraction angle 2*θ*. Purple and red line are the modulations of 30 and 300 K. The modulation at 30 K is almost 10 times smaller than 300 K. Supplementary Fig. S4 shows powder profiles at 30 and 300 K from the first data integrated by 51 pixels. The temperature dependent variation is approximately 1000 counts between 30 and 300 K. The maximum modulation at Bragg peak in 30 K data is less than 500 counts. The ratio of thermal diffuse scattering intensity to Bragg peak intensity is less than 0.001 at 30 K. We ignored thermal diffuse scattering based on this estimation.

### The estimation of anharmonic thermal vibration

Supplementary Fig. S5 shows the temperature dependence of anharmonic thermal parameters by XD2016^14^ for 30, 100, 200, and 300 K. Anisotropic atomic displacement parameters, *U*_11_ = *U*_22_ = *U*_33_, anharmonic thermal parameters, *D*_1111_ = *D*_2222_ = *D*_3333_ and *D*_1122_ = *D*_2233_ = *D*_1133_, were refined in the analyses. The reliability factor of the multipole refinement *R*_F_ were 1.98%, 1.85%, 2.49%, and 2.59% for 30, 100, 200, and 300 K, respectively. The *D*_1111_ at less than 200 K was negative indicating no anharmonic thermal vibration below 200 K.

### Extraction of experimental structure factors with multipole modeling

We performed multipole refinement of the observed structure factors extracted from the results of Rietveld refinement. Multipole refinement updated the calculated structure factors. The intensity ratio of the completely overlapped Bragg reflections was changed by the multipole refinement. Extraction of the observed structure factors was improved by powder diffraction pattern fitting using the structure factors obtained from the multipole refinement. The iterative procedure of the multipole refinement and pattern fitting was conducted 10 times until all the parameters were converged within standard uncertainty. The final observed structure factors at 30 K are listed in Supplementary Table S4.

The experimental multipole parameters shown in the Supplementary Table S3. The radial expansion/contraction parameters of 3*s*^2^3*p*^1^ fitted to theoretical structure factors were employed for the initial parameter of the multipole modeling. Extended Hansen-Coppens multipole model including core deformation term was used for the analysis. Scale factor, *s*, radial expansion/contraction parameters of 3*s*^2^3*p*^1^, *κ*_valence_, anisotropic atomic displacement parameters, *U*_11_ = *U*_22_ = *U*_33_, and multipole parameters, H0, were refined in the analyses. Radial expansion/contraction parameters of inner core electrons 1 *s*^2^ and 2*s*^2^2*p*^6^ were fixed to theoretical values.

## Electronic supplementary material

Supplementary Information

## Data Availability

The data related to this study are available from the corresponding author upon reasonable request.
