# Supplementary material for: Tightly binding valence electron in aluminum observed through X-ray charge density study
Source: Sci Rep. 2018 Aug 10;8:11964. doi: 10.1038/s41598-018-30470-1 (PMC6086884; doi:10.1038/s41598-018-30470-1)
Supplement: Supplementary file 1 — Supplementary Information [file 41598_2018_30470_MOESM1_ESM.docx]

**Supplementary Information**

**Tightly binding valence electron in aluminum observed through X-ray charge density study**

Tomoaki Sasaki^1^, Hidetaka Kasai^1,2^ & Eiji Nishibori^1,2*^

^1^ Graduate School of Pure and Applied Sciences, University of Tsukuba, Tsukuba 305-8571, Japan.

^2^ Faculty of Pure and Applied Sciences and Tsukuba Research Center for Energy Materials Science (TREMS), University of Tsukuba, Tsukuba 305-8571, Japan.

Correspondence and requests for materials should be addressed to:

E.N. (nishibori.eiji.ga@u.tsukuba.ac.jp)

**Supplementary Note.** Comparison of static deformation density between WIEN2k PBE and LSDA. Supplementary Figure S1 shows the present static deformation density of **a** WIEN2k PBE and **b** LSDA in (110) plane. The contour interval is from −0.1 to 0.1 eÅ^−3^ with 0.005 eÅ^−3^ step width. Solid and dotted lines show positive and negative contours, respectively. There are aluminum atoms at four corners. Tetrahedral sites have positive charge density maxima. The contour maps are almost identical each other. The charge density at the peak maxima are 0.03 and 0.025 eÅ^−3^ for WIEN2k PBE and LSDA. The static deformation density of WIEN2k PBE is almost identical to WIEN2k LSDA. Therefore, the WIEN2k PBE were used in main text as the theoretical result.


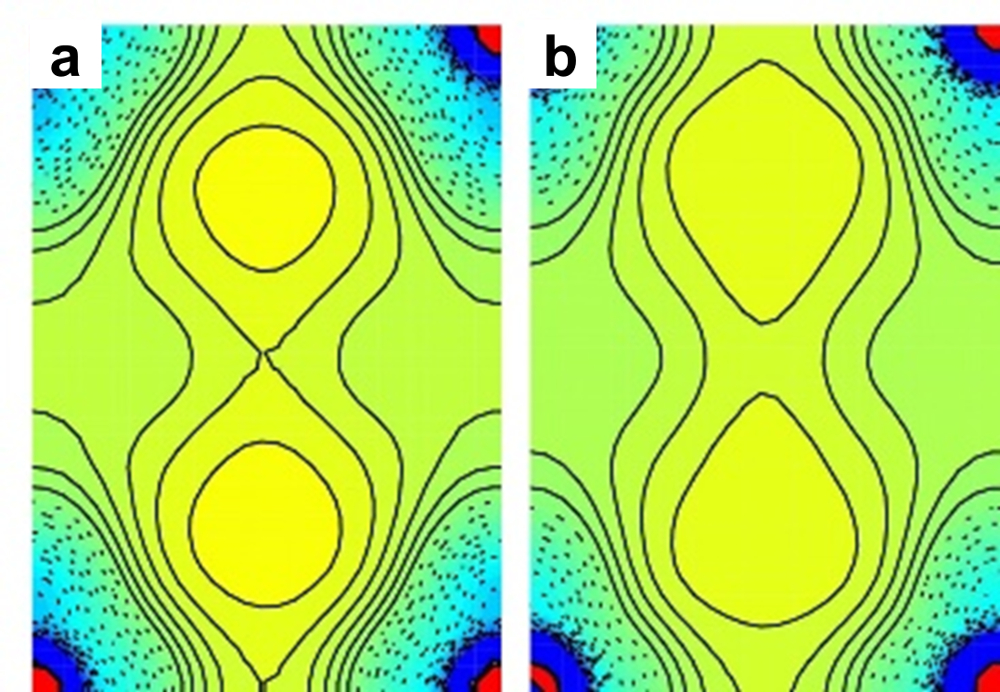


**Supplementary Figure S1.** The present static deformation density of (**a**) WIEN2k PBE and (**b**) LSDA.

**Supplementary Table S1.** The results of structural parameters determined by Rietveld refinement.

|  | 30 K | 100 K | 200 K | 300 K |
| --- | --- | --- | --- | --- |
| *a* (Å) | 4.03270(1) | 4.03318(1) | 4.04117(1) | 4.04940(1) |
| *U*_iso_ (Å^2^) | 0.2284(6) | 0.305(1) | 0.507(3) | 0.731(4) |


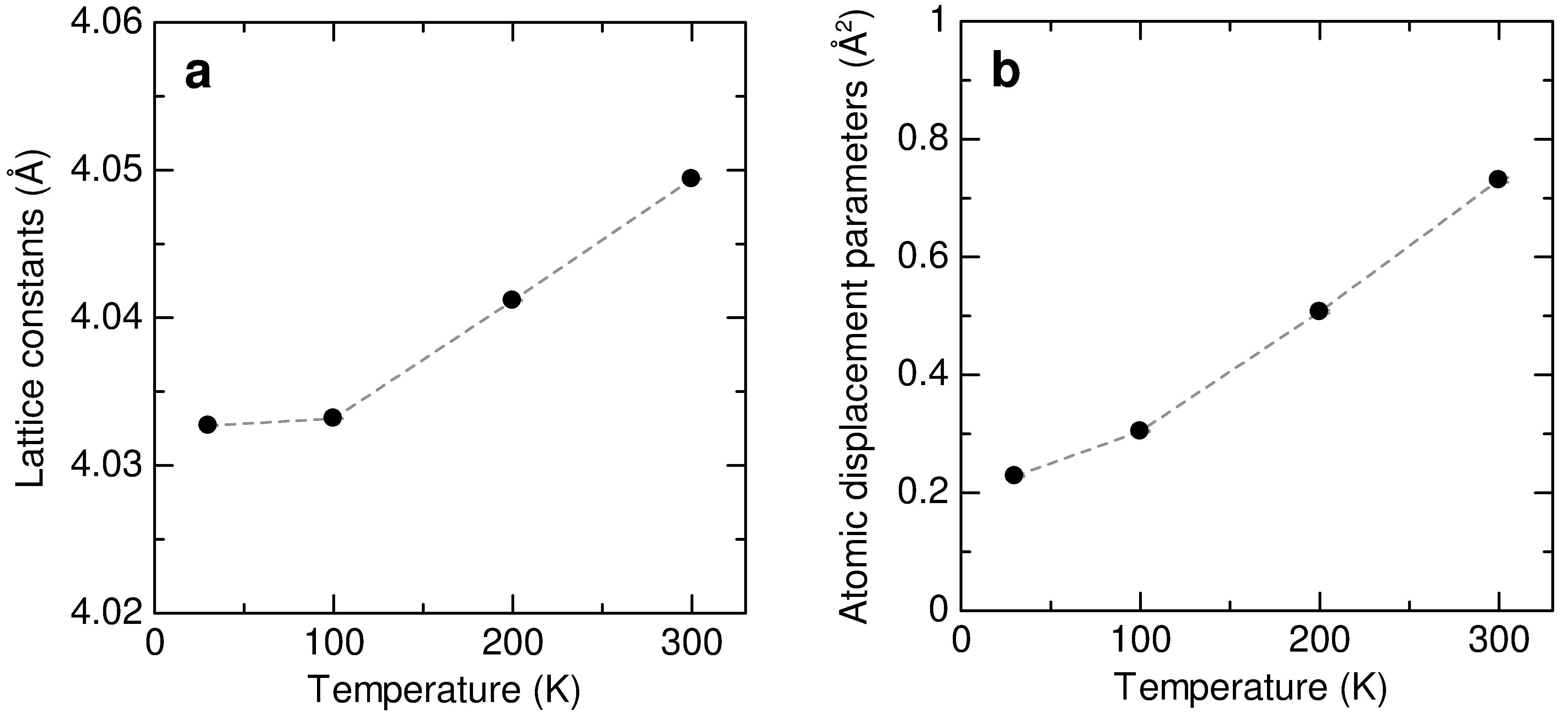


**Supplementary Figure S2.** (**a**) Temperature dependences of lattice constants and (**b**) isotropic atomic displacement parameters.

**Supplementary Table S2.** Theoretical structure factors of PBE and LSDA with sin *θ*/*λ* < 2.31 Å^-1^.

| *h* | *k* | *l* | sin *θ*/*λ* | PBE | LSDA |  | *h* | *k* | *l* | sin *θ*/*λ* | PBE | LSDA |
| --- | --- | --- | --- | --- | --- | --- | --- | --- | --- | --- | --- | --- |
| 1 | 1 | 1 | 0.215 | 8.86 | 8.87 |  | 10 | 4 | 0 | 1.335 | 1.65 | 1.65 |
| 2 | 0 | 0 | 0.248 | 8.38 | 8.39 |  | 10 | 4 | 2 | 1.358 | 1.62 | 1.62 |
| 2 | 2 | 0 | 0.351 | 7.30 | 7.29 |  | 7 | 7 | 5 | 1.375 | 1.60 | 1.61 |
| 3 | 1 | 1 | 0.411 | 6.64 | 6.63 |  | 11 | 1 | 1 | 1.375 | 1.60 | 1.61 |
| 2 | 2 | 2 | 0.430 | 6.45 | 6.43 |  | 8 | 8 | 0 | 1.403 | 1.58 | 1.58 |
| 4 | 0 | 0 | 0.496 | 5.73 | 5.71 |  | 9 | 5 | 5 | 1.419 | 1.56 | 1.56 |
| 3 | 3 | 1 | 0.540 | 5.27 | 5.26 |  | 9 | 7 | 1 | 1.419 | 1.56 | 1.56 |
| 4 | 2 | 0 | 0.554 | 5.13 | 5.12 |  | 11 | 3 | 1 | 1.419 | 1.56 | 1.56 |
| 4 | 2 | 2 | 0.607 | 4.64 | 4.63 |  | 10 | 4 | 4 | 1.424 | 1.55 | 1.55 |
| 3 | 3 | 3 | 0.644 | 4.32 | 4.31 |  | 8 | 8 | 2 | 1.424 | 1.55 | 1.55 |
| 5 | 1 | 1 | 0.644 | 4.32 | 4.31 |  | 8 | 6 | 6 | 1.446 | 1.53 | 1.53 |
| 4 | 4 | 0 | 0.701 | 3.87 | 3.86 |  | 10 | 6 | 0 | 1.446 | 1.53 | 1.53 |
| 5 | 3 | 1 | 0.734 | 3.64 | 3.64 |  | 9 | 7 | 3 | 1.462 | 1.52 | 1.52 |
| 4 | 4 | 2 | 0.744 | 3.57 | 3.57 |  | 11 | 3 | 3 | 1.462 | 1.52 | 1.52 |
| 6 | 0 | 0 | 0.744 | 3.57 | 3.57 |  | 10 | 6 | 2 | 1.467 | 1.51 | 1.51 |
| 6 | 2 | 0 | 0.784 | 3.32 | 3.31 |  | 8 | 8 | 4 | 1.488 | 1.50 | 1.50 |
| 5 | 3 | 3 | 0.813 | 3.15 | 3.15 |  | 12 | 0 | 0 | 1.488 | 1.50 | 1.50 |
| 6 | 2 | 2 | 0.822 | 3.10 | 3.10 |  | 7 | 7 | 7 | 1.503 | 1.48 | 1.48 |
| 4 | 4 | 4 | 0.859 | 2.91 | 2.91 |  | 11 | 5 | 1 | 1.503 | 1.48 | 1.48 |
| 5 | 5 | 1 | 0.885 | 2.79 | 2.78 |  | 12 | 2 | 0 | 1.508 | 1.48 | 1.48 |
| 7 | 1 | 1 | 0.885 | 2.79 | 2.78 |  | 10 | 6 | 4 | 1.529 | 1.46 | 1.46 |
| 6 | 4 | 0 | 0.894 | 2.75 | 2.74 |  | 12 | 2 | 2 | 1.529 | 1.46 | 1.46 |
| 6 | 4 | 2 | 0.928 | 2.60 | 2.60 |  | 9 | 7 | 5 | 1.544 | 1.45 | 1.45 |
| 5 | 5 | 3 | 0.952 | 2.51 | 2.51 |  | 11 | 5 | 3 | 1.544 | 1.45 | 1.45 |
| 7 | 3 | 1 | 0.952 | 2.51 | 2.51 |  | 12 | 4 | 0 | 1.568 | 1.43 | 1.43 |
| 8 | 0 | 0 | 0.992 | 2.37 | 2.37 |  | 9 | 9 | 1 | 1.583 | 1.42 | 1.42 |
| 7 | 3 | 3 | 1.015 | 2.29 | 2.29 |  | 8 | 8 | 6 | 1.588 | 1.42 | 1.42 |
| 6 | 4 | 4 | 1.022 | 2.27 | 2.27 |  | 10 | 8 | 0 | 1.588 | 1.42 | 1.42 |
| 8 | 2 | 0 | 1.022 | 2.27 | 2.27 |  | 12 | 4 | 2 | 1.588 | 1.42 | 1.42 |
| 6 | 6 | 0 | 1.052 | 2.18 | 2.18 |  | 10 | 8 | 2 | 1.607 | 1.41 | 1.41 |
| 8 | 2 | 2 | 1.052 | 2.18 | 2.18 |  | 9 | 9 | 3 | 1.621 | 1.40 | 1.40 |
| 5 | 5 | 5 | 1.074 | 2.13 | 2.13 |  | 11 | 5 | 5 | 1.621 | 1.40 | 1.40 |
| 7 | 5 | 1 | 1.074 | 2.13 | 2.13 |  | 11 | 7 | 1 | 1.621 | 1.40 | 1.40 |
| 6 | 6 | 2 | 1.081 | 2.11 | 2.11 |  | 13 | 1 | 1 | 1.621 | 1.40 | 1.40 |
| 8 | 4 | 0 | 1.109 | 2.04 | 2.04 |  | 10 | 6 | 6 | 1.626 | 1.39 | 1.39 |
| 7 | 5 | 3 | 1.130 | 1.99 | 1.99 |  | 12 | 4 | 4 | 1.645 | 1.38 | 1.38 |
| 9 | 1 | 1 | 1.130 | 1.99 | 1.99 |  | 9 | 7 | 7 | 1.659 | 1.37 | 1.37 |
| 8 | 4 | 2 | 1.136 | 1.98 | 1.98 |  | 11 | 7 | 3 | 1.659 | 1.37 | 1.37 |
| 6 | 6 | 4 | 1.163 | 1.92 | 1.92 |  | 13 | 3 | 1 | 1.659 | 1.37 | 1.37 |
| 9 | 3 | 1 | 1.183 | 1.88 | 1.88 |  | 10 | 8 | 4 | 1.663 | 1.37 | 1.37 |
| 8 | 4 | 4 | 1.215 | 1.83 | 1.83 |  | 12 | 6 | 0 | 1.663 | 1.37 | 1.37 |
| 7 | 5 | 5 | 1.234 | 1.79 | 1.79 |  | 12 | 6 | 2 | 1.682 | 1.36 | 1.36 |
| 7 | 7 | 1 | 1.234 | 1.79 | 1.79 |  | 9 | 9 | 5 | 1.695 | 1.35 | 1.35 |
| 9 | 3 | 3 | 1.234 | 1.79 | 1.79 |  | 13 | 3 | 3 | 1.695 | 1.35 | 1.35 |
| 8 | 6 | 0 | 1.240 | 1.78 | 1.78 |  | 8 | 8 | 8 | 1.718 | 1.34 | 1.34 |
| 10 | 0 | 0 | 1.240 | 1.78 | 1.78 |  | 11 | 7 | 5 | 1.731 | 1.33 | 1.33 |
| 8 | 6 | 2 | 1.264 | 1.75 | 1.75 |  | 13 | 5 | 1 | 1.731 | 1.33 | 1.33 |
| 10 | 2 | 0 | 1.264 | 1.75 | 1.75 |  | 12 | 6 | 4 | 1.736 | 1.33 | 1.33 |
| 7 | 7 | 3 | 1.283 | 1.72 | 1.72 |  | 14 | 0 | 0 | 1.736 | 1.33 | 1.33 |
| 9 | 5 | 1 | 1.283 | 1.72 | 1.72 |  | 10 | 8 | 6 | 1.753 | 1.32 | 1.32 |
| 6 | 6 | 6 | 1.289 | 1.71 | 1.71 |  | 10 | 10 | 0 | 1.753 | 1.32 | 1.32 |
| 10 | 2 | 2 | 1.289 | 1.71 | 1.71 |  | 14 | 2 | 0 | 1.753 | 1.32 | 1.32 |
| 9 | 5 | 3 | 1.330 | 1.66 | 1.66 |  | 11 | 9 | 1 | 1.767 | 1.31 | 1.31 |
| 8 | 6 | 4 | 1.335 | 1.65 | 1.65 |  | 13 | 5 | 3 | 1.767 | 1.31 | 1.31 |
| 10 | 10 | 2 | 1.771 | 1.31 | 1.31 |  | 11 | 9 | 9 | 2.086 | 1.16 | 1.15 |
| 14 | 2 | 2 | 1.771 | 1.31 | 1.31 |  | 15 | 7 | 3 | 2.086 | 1.16 | 1.15 |
| 12 | 8 | 0 | 1.788 | 1.30 | 1.30 |  | 12 | 12 | 0 | 2.104 | 1.15 | 1.14 |
| 9 | 9 | 7 | 1.801 | 1.29 | 1.29 |  | 16 | 4 | 4 | 2.104 | 1.15 | 1.14 |
| 11 | 9 | 3 | 1.801 | 1.29 | 1.29 |  | 11 | 11 | 7 | 2.115 | 1.14 | 1.14 |
| 12 | 8 | 2 | 1.805 | 1.29 | 1.29 |  | 13 | 11 | 1 | 2.115 | 1.14 | 1.14 |
| 14 | 4 | 0 | 1.805 | 1.29 | 1.29 |  | 17 | 1 | 1 | 2.115 | 1.14 | 1.14 |
| 10 | 10 | 4 | 1.822 | 1.28 | 1.28 |  | 12 | 12 | 2 | 2.119 | 1.14 | 1.14 |
| 12 | 6 | 6 | 1.822 | 1.28 | 1.28 |  | 16 | 6 | 0 | 2.119 | 1.14 | 1.14 |
| 14 | 4 | 2 | 1.822 | 1.28 | 1.28 |  | 14 | 8 | 6 | 2.133 | 1.13 | 1.13 |
| 11 | 7 | 7 | 1.835 | 1.27 | 1.27 |  | 16 | 6 | 2 | 2.133 | 1.13 | 1.13 |
| 13 | 5 | 5 | 1.835 | 1.27 | 1.27 |  | 14 | 10 | 0 | 2.133 | 1.13 | 1.13 |
| 13 | 7 | 1 | 1.835 | 1.27 | 1.27 |  | 13 | 9 | 7 | 2.144 | 1.13 | 1.13 |
| 12 | 8 | 4 | 1.856 | 1.26 | 1.26 |  | 13 | 11 | 3 | 2.144 | 1.13 | 1.13 |
| 11 | 9 | 5 | 1.868 | 1.26 | 1.26 |  | 15 | 7 | 5 | 2.144 | 1.13 | 1.13 |
| 13 | 7 | 3 | 1.868 | 1.26 | 1.26 |  | 17 | 3 | 1 | 2.144 | 1.13 | 1.13 |
| 15 | 1 | 1 | 1.868 | 1.26 | 1.26 |  | 10 | 10 | 10 | 2.148 | 1.13 | 1.12 |
| 10 | 8 | 8 | 1.872 | 1.26 | 1.25 |  | 14 | 10 | 2 | 2.148 | 1.13 | 1.12 |
| 14 | 4 | 4 | 1.872 | 1.26 | 1.25 |  | 12 | 12 | 4 | 2.162 | 1.12 | 1.12 |
| 14 | 6 | 0 | 1.889 | 1.25 | 1.25 |  | 15 | 9 | 1 | 2.172 | 1.12 | 1.11 |
| 15 | 3 | 1 | 1.901 | 1.24 | 1.24 |  | 17 | 3 | 3 | 2.172 | 1.12 | 1.11 |
| 10 | 10 | 6 | 1.905 | 1.24 | 1.24 |  | 12 | 10 | 8 | 2.176 | 1.11 | 1.11 |
| 14 | 6 | 2 | 1.905 | 1.24 | 1.24 |  | 16 | 6 | 4 | 2.176 | 1.11 | 1.11 |
| 9 | 9 | 9 | 1.933 | 1.23 | 1.22 |  | 14 | 10 | 4 | 2.190 | 1.11 | 1.10 |
| 13 | 7 | 5 | 1.933 | 1.23 | 1.22 |  | 13 | 11 | 5 | 2.201 | 1.10 | 1.10 |
| 11 | 11 | 1 | 1.933 | 1.23 | 1.22 |  | 15 | 9 | 3 | 2.201 | 1.10 | 1.10 |
| 15 | 3 | 3 | 1.933 | 1.23 | 1.22 |  | 17 | 5 | 1 | 2.201 | 1.10 | 1.10 |
| 12 | 8 | 6 | 1.937 | 1.22 | 1.22 |  | 16 | 8 | 0 | 2.218 | 1.10 | 1.09 |
| 12 | 10 | 0 | 1.937 | 1.22 | 1.22 |  | 11 | 11 | 9 | 2.228 | 1.09 | 1.09 |
| 12 | 10 | 2 | 1.953 | 1.22 | 1.21 |  | 15 | 7 | 7 | 2.228 | 1.09 | 1.09 |
| 14 | 6 | 4 | 1.953 | 1.22 | 1.21 |  | 17 | 5 | 3 | 2.228 | 1.09 | 1.09 |
| 11 | 9 | 7 | 1.964 | 1.21 | 1.21 |  | 12 | 12 | 6 | 2.232 | 1.09 | 1.09 |
| 11 | 11 | 3 | 1.964 | 1.21 | 1.21 |  | 14 | 8 | 8 | 2.232 | 1.09 | 1.09 |
| 13 | 9 | 1 | 1.964 | 1.21 | 1.21 |  | 16 | 8 | 2 | 2.232 | 1.09 | 1.09 |
| 15 | 5 | 1 | 1.964 | 1.21 | 1.21 |  | 18 | 0 | 0 | 2.232 | 1.09 | 1.09 |
| 16 | 0 | 0 | 1.984 | 1.20 | 1.20 |  | 16 | 6 | 6 | 2.245 | 1.08 | 1.08 |
| 13 | 9 | 3 | 1.995 | 1.20 | 1.19 |  | 18 | 2 | 0 | 2.245 | 1.08 | 1.08 |
| 15 | 5 | 3 | 1.995 | 1.20 | 1.19 |  | 13 | 9 | 9 | 2.256 | 1.08 | 1.08 |
| 12 | 10 | 4 | 1.999 | 1.19 | 1.19 |  | 15 | 9 | 5 | 2.256 | 1.08 | 1.08 |
| 14 | 8 | 0 | 1.999 | 1.19 | 1.19 |  | 14 | 10 | 6 | 2.259 | 1.08 | 1.07 |
| 16 | 2 | 0 | 1.999 | 1.19 | 1.19 |  | 18 | 2 | 2 | 2.259 | 1.08 | 1.07 |
| 10 | 10 | 8 | 2.015 | 1.19 | 1.18 |  | 16 | 8 | 4 | 2.273 | 1.07 | 1.07 |
| 14 | 8 | 2 | 2.015 | 1.19 | 1.18 |  | 13 | 11 | 7 | 2.283 | 1.07 | 1.06 |
| 16 | 2 | 2 | 2.015 | 1.19 | 1.18 |  | 17 | 5 | 5 | 2.283 | 1.07 | 1.06 |
| 11 | 11 | 5 | 2.026 | 1.18 | 1.18 |  | 13 | 13 | 1 | 2.283 | 1.07 | 1.06 |
| 13 | 7 | 7 | 2.026 | 1.18 | 1.18 |  | 17 | 7 | 1 | 2.283 | 1.07 | 1.06 |
| 14 | 6 | 6 | 2.030 | 1.18 | 1.18 |  | 14 | 12 | 0 | 2.286 | 1.07 | 1.06 |
| 12 | 8 | 8 | 2.045 | 1.17 | 1.17 |  | 18 | 4 | 0 | 2.286 | 1.07 | 1.06 |
| 16 | 4 | 0 | 2.045 | 1.17 | 1.17 |  | 12 | 10 | 10 | 2.300 | 1.06 | 1.06 |
| 13 | 9 | 5 | 2.056 | 1.17 | 1.17 |  | 14 | 12 | 2 | 2.300 | 1.06 | 1.06 |
| 15 | 5 | 5 | 2.056 | 1.17 | 1.17 |  | 18 | 4 | 2 | 2.300 | 1.06 | 1.06 |
| 15 | 7 | 1 | 2.056 | 1.17 | 1.17 |  | 13 | 13 | 3 | 2.310 | 1.06 | 1.05 |
| 14 | 8 | 4 | 2.060 | 1.17 | 1.16 |  | 17 | 7 | 3 | 2.310 | 1.06 | 1.05 |
| 16 | 4 | 2 | 2.060 | 1.17 | 1.16 |  | 15 | 11 | 1 | 2.310 | 1.06 | 1.05 |
| 12 | 10 | 6 | 2.075 | 1.16 | 1.16 |  |  |  |  |  |  |  |

**Supplementary Table S3.** Multipole parameters for the theoretical structure factors of PBE, LSDA, and the experimental structure factors at 30 K.

|  | PBE | LSDA | 30 K |
| --- | --- | --- | --- |
| *R*_F_ / *Rw*_F_ (%) | 0.08 / 0.1 | 0.1 / 0.11 | 1.97 / 1.27 |
| GOF | 0.1270 | 0.1412 | 1.3321 |
| Scale | 0.75 | 0.75 | 1.006(1) |
| *κ* (1*s*) | 0.9848(8) | 0.9746(9) | 0.9848 |
| *κ* (2*s*2*p*) | 1.0010(3) | 0.9983(3) | 1.0010 |
| *κ* (3*s*3*p*) | 0.943(2) | 0.960(2) | 0.95(2) |
| H0 | -0.00079(4) | -0.00075(5) | -0.0017(5) |
| H4+ | -0.00062(3) | -0.00059(4) | -0.0013(4) |


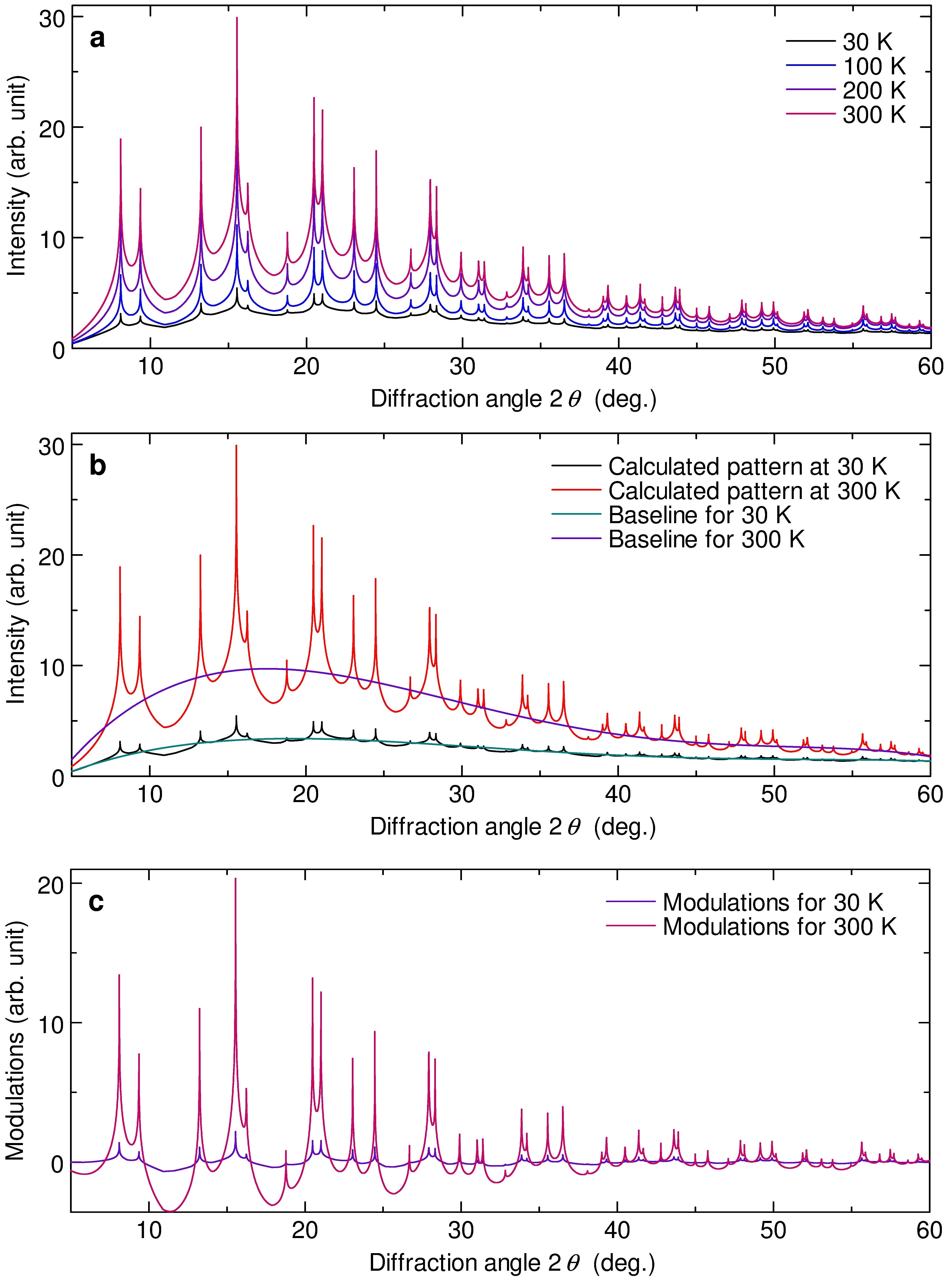


**Supplementary Figure S3.** The first order thermal diffuse scattering and structure factor modulations of aluminum. (**a**) The first order thermal diffuse scattering of aluminum for 30, 100, 200, 300 K based on Herbstein’s equation. (**b**) The first order thermal diffuse scattering of aluminum at 30 and 300 K together with purple and green guided spline curves. (**c**) The modulations of calculated first order thermal diffuse scattering from the baselines.


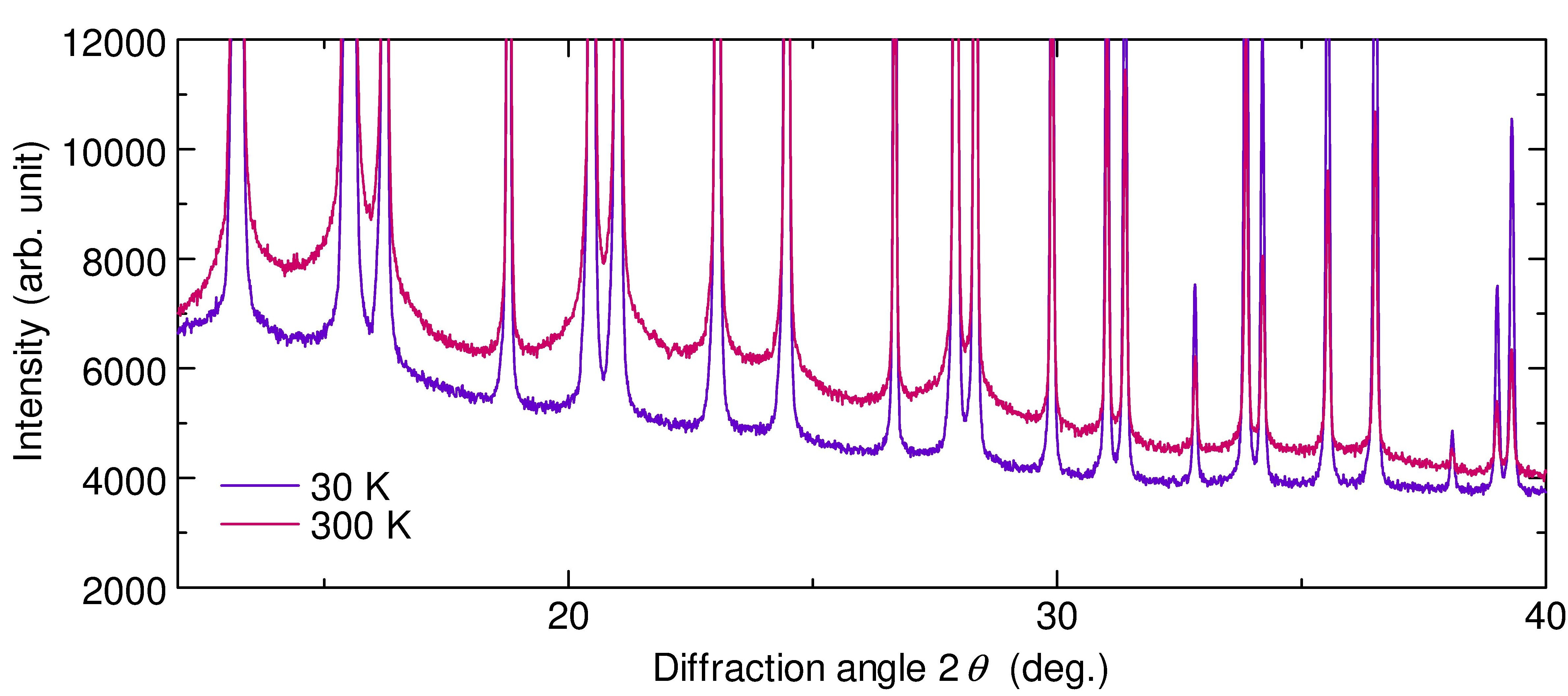


**Supplementary Figure S4.** The present 1-dimensional X-ray diffraction data at 30 and 300 K.


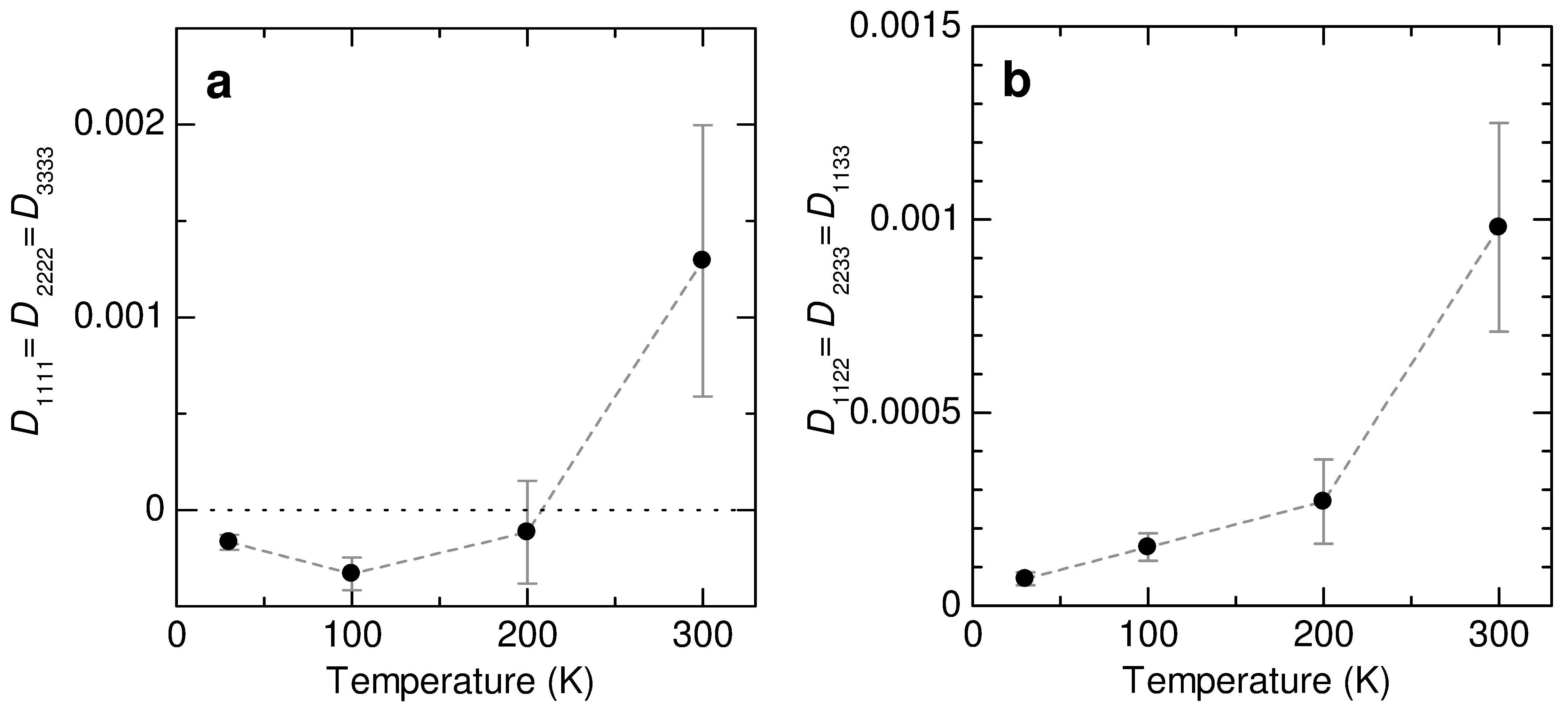


**Supplementary Figure S5.** The temperature dependence of anharmonic thermal parameters (**a**) *D*_1111_ and (**b**) *D*_1122_ for experimental 30, 100, 200, and 300 K data.

**Supplementary Table S4.** The observed structure factors at 30 K with sin *θ*/*λ* < 2.31 Å^-1^.

| *h* | *k* | *l* | sin *θ*/*λ* | 30 K |  | *h* | *k* | *l* | sin *θ*/*λ* | 30 K |  |
| --- | --- | --- | --- | --- | --- | --- | --- | --- | --- | --- | --- |
| 1 | 1 | 1 | 0.215 | 8.91(1) |  | 10 | 4 | 0 | 1.335 | 1.62(3) |  |
| 2 | 0 | 0 | 0.248 | 8.36(1) |  | 10 | 4 | 2 | 1.358 | 1.61(2) |  |
| 2 | 2 | 0 | 0.351 | 7.30(1) |  | 7 | 7 | 5 | 1.375 | 1.59(3) |  |
| 3 | 1 | 1 | 0.411 | 6.62(1) |  | 11 | 1 | 1 | 1.375 | 1.59(3) |  |
| 2 | 2 | 2 | 0.430 | 6.48(1) |  | 8 | 8 | 0 | 1.403 | 1.67(4) |  |
| 4 | 0 | 0 | 0.496 | 5.75(2) |  | 9 | 5 | 5 | 1.419 | 1.54(3) |  |
| 3 | 3 | 1 | 0.540 | 5.23(1) |  | 9 | 7 | 1 | 1.419 | 1.54(2) |  |
| 4 | 2 | 0 | 0.554 | 5.14(1) |  | 11 | 3 | 1 | 1.419 | 1.54(2) |  |
| 4 | 2 | 2 | 0.607 | 4.64(1) |  | 10 | 4 | 4 | 1.424 | 1.54(3) |  |
| 3 | 3 | 3 | 0.644 | 4.35(2) |  | 8 | 8 | 2 | 1.424 | 1.54(3) |  |
| 5 | 1 | 1 | 0.644 | 4.34(1) |  | 8 | 6 | 6 | 1.446 | 1.52(3) |  |
| 4 | 4 | 0 | 0.701 | 3.93(2) |  | 10 | 6 | 0 | 1.446 | 1.52(3) |  |
| 5 | 3 | 1 | 0.734 | 3.63(1) |  | 9 | 7 | 3 | 1.462 | 1.48(2) |  |
| 4 | 4 | 2 | 0.744 | 3.58(1) |  | 11 | 3 | 3 | 1.462 | 1.48(3) |  |
| 6 | 0 | 0 | 0.744 | 3.56(2) |  | 10 | 6 | 2 | 1.467 | 1.48(2) |  |
| 6 | 2 | 0 | 0.784 | 3.28(1) |  | 8 | 8 | 4 | 1.488 | 1.49(3) |  |
| 5 | 3 | 3 | 0.813 | 3.12(1) |  | 12 | 0 | 0 | 1.488 | 1.49(7) |  |
| 6 | 2 | 2 | 0.822 | 3.04(1) |  | 7 | 7 | 7 | 1.503 | 1.46(6) |  |
| 4 | 4 | 4 | 0.859 | 2.91(3) |  | 11 | 5 | 1 | 1.503 | 1.47(2) |  |
| 5 | 5 | 1 | 0.885 | 2.77(2) |  | 12 | 2 | 0 | 1.508 | 1.43(3) |  |
| 7 | 1 | 1 | 0.885 | 2.77(2) |  | 10 | 6 | 4 | 1.529 | 1.44(2) |  |
| 6 | 4 | 0 | 0.894 | 2.72(2) |  | 12 | 2 | 2 | 1.529 | 1.44(4) |  |
| 6 | 4 | 2 | 0.928 | 2.58(1) |  | 9 | 7 | 5 | 1.544 | 1.45(3) |  |
| 5 | 5 | 3 | 0.952 | 2.49(2) |  | 11 | 5 | 3 | 1.544 | 1.45(3) |  |
| 7 | 3 | 1 | 0.952 | 2.49(1) |  | 12 | 4 | 0 | 1.568 | 1.48(4) |  |
| 8 | 0 | 0 | 0.992 | 2.36(4) |  | 9 | 9 | 1 | 1.583 | 1.46(4) |  |
| 7 | 3 | 3 | 1.015 | 2.25(2) |  | 8 | 8 | 6 | 1.588 | 1.42(4) |  |
| 6 | 4 | 4 | 1.022 | 2.22(2) |  | 10 | 8 | 0 | 1.588 | 1.42(4) |  |
| 8 | 2 | 0 | 1.022 | 2.22(2) |  | 12 | 4 | 2 | 1.588 | 1.42(3) |  |
| 6 | 6 | 0 | 1.052 | 2.15(3) |  | 10 | 8 | 2 | 1.607 | 1.45(3) |  |
| 8 | 2 | 2 | 1.052 | 2.15(2) |  | 9 | 9 | 3 | 1.621 | 1.36(4) |  |
| 5 | 5 | 5 | 1.074 | 2.09(3) |  | 11 | 5 | 5 | 1.621 | 1.36(4) |  |
| 7 | 5 | 1 | 1.074 | 2.10(1) |  | 11 | 7 | 1 | 1.621 | 1.37(3) |  |
| 6 | 6 | 2 | 1.081 | 2.05(2) |  | 13 | 1 | 1 | 1.621 | 1.36(4) |  |
| 8 | 4 | 0 | 1.109 | 2.09(2) |  | 10 | 6 | 6 | 1.626 | 1.41(4) |  |
| 7 | 5 | 3 | 1.130 | 1.97(2) |  | 12 | 4 | 4 | 1.645 | 1.44(4) |  |
| 9 | 1 | 1 | 1.130 | 1.97(2) |  | 9 | 7 | 7 | 1.659 | 1.32(4) |  |
| 8 | 4 | 2 | 1.136 | 1.96(2) |  | 11 | 7 | 3 | 1.659 | 1.33(3) |  |
| 6 | 6 | 4 | 1.163 | 1.95(2) |  | 13 | 3 | 1 | 1.659 | 1.33(3) |  |
| 9 | 3 | 1 | 1.183 | 1.89(2) |  | 10 | 8 | 4 | 1.663 | 1.32(3) |  |
| 8 | 4 | 4 | 1.215 | 1.84(2) |  | 12 | 6 | 0 | 1.663 | 1.32(4) |  |
| 7 | 5 | 5 | 1.234 | 1.77(2) |  | 12 | 6 | 2 | 1.682 | 1.33(3) |  |
| 7 | 7 | 1 | 1.234 | 1.76(2) |  | 9 | 9 | 5 | 1.695 | 1.44(4) |  |
| 9 | 3 | 3 | 1.234 | 1.76(2) |  | 13 | 3 | 3 | 1.695 | 1.44(4) |  |
| 8 | 6 | 0 | 1.240 | 1.75(2) |  | 8 | 8 | 8 | 1.718 | 1.67(8) |  |
| 10 | 0 | 0 | 1.240 | 1.75(5) |  | 11 | 7 | 5 | 1.731 | 1.30(3) |  |
| 8 | 6 | 2 | 1.264 | 1.73(2) |  | 13 | 5 | 1 | 1.731 | 1.30(3) |  |
| 10 | 2 | 0 | 1.264 | 1.73(3) |  | 12 | 6 | 4 | 1.736 | 1.30(3) |  |
| 7 | 7 | 3 | 1.283 | 1.70(3) |  | 14 | 0 | 0 | 1.736 | 1.28(9) |  |
| 9 | 5 | 1 | 1.283 | 1.70(2) |  | 10 | 8 | 6 | 1.753 | 1.28(3) |  |
| 6 | 6 | 6 | 1.289 | 1.67(5) |  | 10 | 10 | 0 | 1.753 | 1.27(7) |  |
| 10 | 2 | 2 | 1.289 | 1.68(3) |  | 14 | 2 | 0 | 1.753 | 1.28(5) |  |
| 9 | 5 | 3 | 1.330 | 1.66(2) |  | 11 | 9 | 1 | 1.767 | 1.25(3) |  |
| 8 | 6 | 4 | 1.335 | 1.62(2) |  | 13 | 5 | 3 | 1.767 | 1.25(3) |  |
| 10 | 10 | 2 | 1.771 | 1.29(5) |  | 11 | 9 | 9 | 2.086 | 1.20(7) | |
| 14 | 2 | 2 | 1.771 | 1.29(5) |  | 15 | 7 | 3 | 2.086 | 1.21(5) | |
| 12 | 8 | 0 | 1.788 | 1.35(5) |  | 12 | 12 | 0 | 2.104 | 1.2(1) | |
| 9 | 9 | 7 | 1.801 | 1.25(5) |  | 16 | 4 | 4 | 2.104 | 1.22(7) | |
| 11 | 9 | 3 | 1.801 | 1.25(3) |  | 11 | 11 | 7 | 2.115 | 0.95(7) | |
| 12 | 8 | 2 | 1.805 | 1.27(4) |  | 13 | 11 | 1 | 2.115 | 0.96(5) | |
| 14 | 4 | 0 | 1.805 | 1.27(5) |  | 17 | 1 | 1 | 2.115 | 0.95(7) | |
| 10 | 10 | 4 | 1.822 | 1.28(5) |  | 12 | 12 | 2 | 2.119 | 1.02(7) | |
| 12 | 6 | 6 | 1.822 | 1.28(5) |  | 16 | 6 | 0 | 2.119 | 1.02(7) | |
| 14 | 4 | 2 | 1.822 | 1.28(4) |  | 14 | 8 | 6 | 2.133 | 1.07(5) | |
| 11 | 7 | 7 | 1.835 | 1.21(5) |  | 16 | 6 | 2 | 2.133 | 1.07(5) | |
| 13 | 5 | 5 | 1.835 | 1.21(5) |  | 14 | 10 | 0 | 2.133 | 1.06(7) | |
| 13 | 7 | 1 | 1.835 | 1.22(4) |  | 13 | 9 | 7 | 2.144 | 1.09(5) | |
| 12 | 8 | 4 | 1.856 | 1.22(4) |  | 13 | 11 | 3 | 2.144 | 1.09(5) | |
| 11 | 9 | 5 | 1.868 | 1.23(4) |  | 15 | 7 | 5 | 2.144 | 1.09(5) | |
| 13 | 7 | 3 | 1.868 | 1.23(4) |  | 17 | 3 | 1 | 2.144 | 1.09(5) | |
| 15 | 1 | 1 | 1.868 | 1.22(5) |  | 10 | 10 | 10 | 2.148 | 1.2(1) | |
| 10 | 8 | 8 | 1.872 | 1.27(5) |  | 14 | 10 | 2 | 2.148 | 1.25(5) | |
| 14 | 4 | 4 | 1.872 | 1.27(5) |  | 12 | 12 | 4 | 2.162 | 1.67(8) | |
| 14 | 6 | 0 | 1.889 | 1.32(5) |  | 15 | 9 | 1 | 2.172 | 1.14(5) | |
| 15 | 3 | 1 | 1.901 | 1.20(4) |  | 17 | 3 | 3 | 2.172 | 1.14(8) | |
| 10 | 10 | 6 | 1.905 | 1.26(6) |  | 12 | 10 | 8 | 2.176 | 1.19(6) | |
| 14 | 6 | 2 | 1.905 | 1.27(4) |  | 16 | 6 | 4 | 2.176 | 1.19(6) | |
| 9 | 9 | 9 | 1.933 | 1.3(1) |  | 14 | 10 | 4 | 2.190 | 1.18(6) | |
| 13 | 7 | 5 | 1.933 | 1.28(4) |  | 13 | 11 | 5 | 2.201 | 1.02(6) | |
| 11 | 11 | 1 | 1.933 | 1.27(6) |  | 15 | 9 | 3 | 2.201 | 1.02(6) | |
| 15 | 3 | 3 | 1.933 | 1.27(6) |  | 17 | 5 | 1 | 2.201 | 1.02(6) | |
| 12 | 8 | 6 | 1.937 | 1.31(4) |  | 16 | 8 | 0 | 2.218 | 1.06(8) | |
| 12 | 10 | 0 | 1.937 | 1.30(6) |  | 11 | 11 | 9 | 2.228 | 0.96(8) | |
| 12 | 10 | 2 | 1.953 | 1.18(4) |  | 15 | 7 | 7 | 2.228 | 0.96(8) | |
| 14 | 6 | 4 | 1.953 | 1.18(4) |  | 17 | 5 | 3 | 2.228 | 0.97(6) | |
| 11 | 9 | 7 | 1.964 | 1.18(4) |  | 12 | 12 | 6 | 2.232 | 1.06(8) | |
| 11 | 11 | 3 | 1.964 | 1.16(6) |  | 14 | 8 | 8 | 2.232 | 1.06(8) | |
| 13 | 9 | 1 | 1.964 | 1.18(4) |  | 16 | 8 | 2 | 2.232 | 1.07(6) | |
| 15 | 5 | 1 | 1.964 | 1.18(4) |  | 18 | 0 | 0 | 2.232 | 1.1(2) | |
| 16 | 0 | 0 | 1.984 | 1.7(1) |  | 16 | 6 | 6 | 2.245 | 1.26(8) | |
| 13 | 9 | 3 | 1.995 | 1.11(4) |  | 18 | 2 | 0 | 2.245 | 1.26(8) | |
| 15 | 5 | 3 | 1.995 | 1.11(4) |  | 13 | 9 | 9 | 2.256 | 1.13(9) | |
| 12 | 10 | 4 | 1.999 | 1.18(4) |  | 15 | 9 | 5 | 2.256 | 1.13(6) | |
| 14 | 8 | 0 | 1.999 | 1.17(6) |  | 14 | 10 | 6 | 2.259 | 1.18(6) | |
| 16 | 2 | 0 | 1.999 | 1.17(6) |  | 18 | 2 | 2 | 2.259 | 1.18(9) | |
| 10 | 10 | 8 | 2.015 | 1.15(6) |  | 16 | 8 | 4 | 2.273 | 1.18(6) | |
| 14 | 8 | 2 | 2.015 | 1.16(5) |  | 13 | 11 | 7 | 2.283 | 0.99(6) | |
| 16 | 2 | 2 | 2.015 | 1.15(6) |  | 17 | 5 | 5 | 2.283 | 0.99(9) | |
| 11 | 11 | 5 | 2.026 | 1.29(6) |  | 13 | 13 | 1 | 2.283 | 0.99(9) | |
| 13 | 7 | 7 | 2.026 | 1.29(6) |  | 17 | 7 | 1 | 2.283 | 0.99(6) | |
| 14 | 6 | 6 | 2.030 | 1.39(7) |  | 14 | 12 | 0 | 2.286 | 1.16(9) | |
| 12 | 8 | 8 | 2.045 | 1.29(7) |  | 18 | 4 | 0 | 2.286 | 1.16(9) | |
| 16 | 4 | 0 | 2.045 | 1.29(7) |  | 12 | 10 | 10 | 2.300 | 1.08(9) | |
| 13 | 9 | 5 | 2.056 | 1.07(5) |  | 14 | 12 | 2 | 2.300 | 1.08(6) | |
| 15 | 5 | 5 | 2.056 | 1.06(7) |  | 18 | 4 | 2 | 2.300 | 1.08(6) | |
| 15 | 7 | 1 | 2.056 | 1.07(5) |  | 13 | 13 | 3 | 2.310 | 0.88(9) | |
| 14 | 8 | 4 | 2.060 | 1.15(5) |  | 17 | 7 | 3 | 2.310 | 0.88(6) | |
| 16 | 4 | 2 | 2.060 | 1.15(5) |  | 15 | 11 | 1 | 2.310 | 0.88(6) | |
| 12 | 10 | 6 | 2.075 | 1.23(5) |  |  |  |  |  |  | |
